# CD105+ fibroblasts support an immunosuppressive niche in women at high risk of breast cancer initiation

**DOI:** 10.21203/rs.3.rs-5777126/v1

**Published:** 2025-04-02

**Authors:** Eric G. Carlson, Jennifer C. Lopez, Yukiko Yamaguchi, Jackson Gibson, Saul Priceman, Mark A. LaBarge

## Abstract

Background: Aging is the greatest risk factor for breast cancer, and although epithelial cells are the source of carcinomas, epithelial changes alone do not fully explain cancer susceptibility. Fibroblasts and macrophages are key stromal constituents around the cells of origin for cancer in breast tissue. With age, macrophages surrounding terminal ductal lobular units (TDLUs) become increasingly immunosuppressive. CD105 ^+^ fibroblasts intercalate within TDLUs, drive luminal differentiation, and give rise to immunosuppressive cancer-associated fibroblasts in other tissues. We propose that differences in fibroblasts are a crucial component of the stroma that shapes cancer susceptibility. Methods: Primary fibroblast cultures were established from prophylactic and reduction mammoplasties from women ranging in age from 16 to 70 years and breast cancer risk ( *BRCA1* mutation carriers). Growth characteristics, transcriptional profiles, differentiation potential, and secreted proteins were profiled for fibroblast subtypes from diverse donors. Co-cultures with fibroblasts, monocytes, macrophages, and T cells were used to ascertain the functional role played by CD105 ^+^ fibroblasts in immune cell modulation. Results: We found that peri-epithelial CD105 ^+^ fibroblasts are enriched in older women as well as women who carry *BRCA1* mutations. These CD105 ^+^ fibroblasts exhibit robust adipogenesis and secrete factors related to macrophage polarization. Macrophages cocultured with fibroblasts better maintain or enhance polarization states than media alone. CD105 ^+^ fibroblasts increased expression of immunosuppressive macrophage genes. CD105 ^+^ fibroblasts supported anti-inflammatory macrophage-mediated suppression of T cell proliferation, whereas CD105 ^−^ fibroblasts significantly reduced the suppressive effect of anti-inflammatory macrophages on T cell proliferation. Conclusions: Establishment of a coculture system to dissect the molecular circuits between CD105 ^+^ fibroblasts and macrophages that drive immunosuppressive macrophage polarization has broad utility in understanding mammary gland development and events that precede cancer initiation. CD105 ^+^ fibroblasts and macrophages may coordinate to suppress immunosurveillance and increase breast cancer susceptibility.

## Background

The epithelial structures within the breast responsible for producing milk during lactation are known as terminal ductal lobular units (TDLUs). A TDLU is a collection of acini arising from one terminal duct and is embedded in intralobular stroma, which all together, is considered a functional unit of the breast. These structures are incredibly dynamic and are characterized by extracellular matrix (ECM) and cellular turnover throughout a woman's life (1–4). It is within these highly dynamic structures that breast cancer arises (5, 6).

Understanding how the dynamic microenvironment of the breast contributes to increased susceptibility of breast cancer initiation would improve risk stratification of women and open opportunities for the development of preventative therapeutics. Essential components of the intralobular stroma are fibroblasts and macrophages. The type-1 transmembrane glycoprotein endoglin (CD105, *ENG*) and dipeptidyl peptidase-4 (CD26, *DPP4*) are expressed by fibroblast subpopulations in healthy and diseased breast tissue (7–10). CD26 has been used as a marker for fibroblast subtypes in the mouse mammary gland (11, 12), whereas it is unstable in culture and has weaker protein-level expression when compared to CD105 in humans (8, 13–15). Fibroblasts that express CD105 ($CD105^+$) describe a population that is enriched in the intralobular stroma (8). Macrophages play essential roles in mammary gland homeostasis and development (16–18).They also defend against pathogens, are plastic, and may be categorized according to different functions. Classically activated, or pro-inflammatory, macrophages migrate to inflamed tissues, kill pathogens through the production of reactive oxygen species, and are capable of activating the adaptive immune system (19). Alternatively activated, or anti-inflammatory, macrophages facilitate tissue repair and induce immune tolerance by attracting regulatory T cells (Tregs) and Th2 T cells devoid of cytotoxic function (20). Postmenopausal women have more anti-inflammatory macrophages in their breasts compared to premenopausal women (21) and these anti-inflammatory macrophages preferentially localize to intralobular stroma when compared to their pro-inflammatory counterparts (22). How proportions of fibroblast subtypes change with age and breast cancer risk is an emerging area of interest in understanding cancer susceptibility (23, 24). Additionally, while interactions between fibroblasts and macrophages are well studied in tumor microenvironments (25–28), how these interactions in normal tissue contribute to breast cancer susceptibility is not well understood.

Herein, peri-epithelial fibroblast subtype composition was defined in early passage, finite fibroblast strains from 30 different women to evaluate how fibroblasts change in average risk older women and high-risk women with pathogenic *BRCA1* mutations. Numerous assays were performed to determine differences between $CD105^+$ and $CD105^-$ fibroblasts from different individuals at the transcriptional, protein, and functional levels. Immune cell cocultures were established between fibroblast subtypes and innate and adaptive immune cells. The signature for primary $CD105^+$ fibroblasts in culture was applied to publicly available human breast atlas datasets to ground the relevance of the findings *in vivo*. $CD105^+$ fibroblasts were found to be enriched in cultures established from women at high risk of developing breast cancer, were enriched for pathways related to an inflammatory milieu with the potential for negative regulation of the immune system, exhibited robust adipogenic potential, and were found to influence macrophage polarization both in culture and *in vivo*.

### Methods

## Human subjects

The present study was approved by the Human Subjects Committee and the Institutional Review Board (IRB) 17185 at City of Hope. Twenty-five women were consented in person and sequentially; all women signed a City of Hope IRB-approved consent before trial entry. Women were eligible for the present study if they were undergoing a breast reduction or prophylactic mastectomy. Ten breast organoids from reduction mammoplasties were used to establish fibroblast strains prepared at the Lawrence Berkeley National Laboratory (Berkeley, CA) with approved IRB for sample distribution and collection from specific locations. Metadata including: age, BMI, self-reported ethnicity, family history of cancer, treatment, diagnosis, and *BRCA1* mutational status are included in **Supplementary Table 1**. Three leukapheresis products (whole blood discard kits) were obtained from consented healthy donors under protocols approved by the City of Hope Internal Review Board.

### Isolation of fibroblasts

Peri-epithelial fibroblasts were isolated from primary organoids using differential trypsinization, as described previously (29). Briefly, Human mammary tissue is obtained as discard material from surgical procedures. The epithelial areas are separated from the stromal matrix of adipose tissue, connective tissue, and blood vessels using sterile scalpels. The epithelial tissue is cut into smaller 3–4 mm pieces to aid digestion. The dissected epithelial tissue is placed in a conical centrifuge tube and enzymatically digested in media supplemented with 200 U/ml crude collagenase and 100 U/ml hyaluronidase overnight at 37°C with gentle agitation. The tube is centrifuged to discard supernatant fat and medium. Organoids are resuspended in media. Digestion is complete when microscopic examination of organoids shows presence of ductal, alveolar, or ductal-alveolar structures. Digested organoids are added to a 100 µm cell strainer over a sterile 50 ml tube. Organoids are washed and the organoids that do not pass through the filter are collected and plated (approximately 20–40 organoids seeded per 60 mm dish). Cell migration from the organoids is visible by 24–48 hours, and mitotic outgrowth by 48–72 hours. Differential trypsinization with 0.05% trypsin is used to isolate the peri-epithelial fibroblasts from the outgrowths based on the rapid detachment of fibroblasts from the surface plastic, relative to epithelial cells. The peri-epithelial fibroblasts are collected and frozen for future expansion. Fibroblasts were maintained on 100mm culture dishes (Greiner Cellstar) in M87A medium (30) supplemented with 5% FBS (Gemini Bio-Products). To ensure that changes to CD105 expression were not cell culture artifacts, fibroblasts were maintained at subconfluence and dissociated with 0.05% trypsin for less than 5 minutes for passaging. 0.05% trypsin was found to produce identical CD105 expression patterns as a nonenzymatic dissociation method, 1mM EDTA. Each strain was confirmed to be negative for mycoplasma prior to use.

## Flow cytometry and cell sorting

Fibroblasts were dissociated using 0.05% trypsin or 1mM EDTA and stained on ice with conjugated antibodies for 25 minutes prior analysis on either an Attune (ThermoFisher) or Quanteon (Agilent) flow cytometer. 1 µg/ml of DAPI was added 10 minutes prior to analysis for dead cell exclusion. See **Supplementary Table 2** for the complete list of antibodies used to profile fibroblasts (data for antibodies in gray are not presented in the manuscript; fibroblasts were uniformly negative for these markers). Unstained cells were used to establish positive and negative gates. FlowJo v.10.6.2 (BD Life Sciences) was used to analyze data. GraphPad Prism v.9.3.1 was used to perform simple linear regression, unpaired t tests with Welch's correction, and 2-way ANOVA with Tukey's multiple comparisons test.

Many experiments (growth curve analysis, RNA-sequencing, adipogenesis, osteogenesis, Luminex, and cocultures) presented in this manuscript necessitated the sorting of cultured fibroblasts into $CD105^+$ and $CD105^-$ subpopulations. Fibroblasts were dissociated using 0.05% trypsin and stained on ice with conjugated antibodies targeting CD105 and in some cases CD26 (**Supplementary Table 2**) for 25 minutes, with rinsing, prior to sorting using an Aria III cell sorter (BD Biosciences). 1 µg/ml of DAPI was added 10 minutes prior to sorting for dead cell exclusion. Unstained cells were used to establish positive and negative gates. Forward scatter (FSC) and side scatter (SSC) were used to identify fibroblasts based on size and granularity. Single cells were selected using FSC-area and FSC-height to exclude doublets. DAPI was used to exclude dead cells. Based on gates set by unstained cells, $CD105^+$ and $CD105^-$ fibroblasts were collected into FACs tubes filled with media.

## Growth curve analysis

To initiate the growth curve assay, fibroblasts from the following strains were grown to subconfluency: C073, 169, C023, and C008 (starting passages are listed in **Supplementary Table 1**). Fibroblasts were then sorted into $CD105^+$ and $CD105^-$ subpopulations using an antibody targeting human CD105 (**Supplementary Table 2**) with an ARIA III cell sorter (BD Biosciences).50,000 cells were seeded in triplicate at the start of every passage in a 6-well plate (5,200 cells/$cm^2$) (CytoOne) and cells were counted at the point the dish became subconfluent. Each replicate was counted using a hemocytometer. Total population doublings (PD) were calculated using the equation $PD = \log2(N^{final}/N^{initial})$. N is the number of cells counted. GraphPad Prism v.9.3.1 was used to perform a 2-way ANOVA on the growth curves from fibroblast subtypes.

## Differentiation assays

For adipogenic differentiation sorted $CD105^+$ and $CD105^-$ fibroblasts (**Supplementary Fig. 4**) were seeded at 20,000 cells per well in 12-well plates (5,700 cells/$cm^2$) (Greiner Cellstar) and changed to adipogenic (100nM dexamethasone (Sigma-Aldrich, D1756), 450uM 1-methyl-3-isobutylxanthine (Sigma-Aldrich, I5879), 1µM Rosiglitazone (Cayman, 71740), and 1µg/ml insulin (Sigma-Aldrich, I5500) in DMEM/F12 (Gibco) + 10%FBS (Gemini Bio-Products)) (31, 32) or control media (DMEM/F12 + 10%FBS) once cells reached subconfluence (typically after 4 days). Adipogenic induction was visualized by Oil Red O (ORO) (Sigma-Aldrich O0625) staining after 2 weeks. Cultures were fixed at room temperature for 15 minutes in 4% paraformaldehyde, cells were rinsed with water, washed with 60% isopropanol (IPA) for 5 minutes, and stained with ORO working solution (0.3 mg/ml Oil Red O dye in 60%IPA) for 20 minutes at room temperature. To quantify ORO staining, cells were rinsed with water, washed with 60% IPA for 5 minutes, and 250µl of 100% IPA was added to each well to solubilize ORO staining. 200µl of the IPA:ORO solution was transferred to a microplate (Corning) and absorbance at 492nm was read on a plate reader (100% IPA was used as a blank). GraphPad Prism v.9.3.1 was used to perform paired T-tests.

For osteogenic differentiation, sorted $CD105^+$ and $CD105^-$ fibroblasts were seeded at 20,000 cells/$cm^2$, and changed to osteoblastic (5mM beta-glycerophosphate (Sigma-Aldrich G9422), 283µM L-ascorbic acid (Sigma-Aldrich A92902), 10nM dexamethasone (Sigma-Aldrich D1756), and 10µM 1α,25-dihydroxyvitamin D3 (Sigma-Aldrich D1530) in DMEM/F12 (Gibco) + 10%FBS (Gemini Bio-Products))(31) or control media (DMEM/F12 + 10%FBS) after two days in culture. Alizarin Red staining (Abcam) was used to assess formation of mineralized matrix after 4 weeks as described (31). Induction of differentiation in human bone marrow-derived MSCs (ATCC, PCS-500-012) was used as a positive control in both assays.

## Luminex assay

100,000 viable sorted $CD105^+$ and $CD105^-$ fibroblasts were seeded into 6-well plates (10,400 cells/$cm^2$) (CytoOne) in M87A + 5% FBS (Gemini Bio-Products). Media was changed after 2 days. 48 hours after the media change, supernatant was collected and centrifuged at 2,000X g for 10 minutes at 4°C. Cell and debris-free supernatant was stored at -80°C until analysis in duplicate on a custom 23-plex (LUM, ALDH1A1, MMP-3, IL-8, CD26, CXCL6, CCL2, IL-1β, DKK-1, SPARC, CXCL1, M-CSF, TSP-2, POSTN, FN1, BMP-2, IL-6, IL-33, MCAM, IGFBP-2, IGFBP-7, LIF, SCF) Luminex assay (R&D Systems). Concentrations of each analyte were adjusted based on values from M87A + 5%FBS media alone. Values below the detectable range were replaced with zeroes. Values above the detectable range were replaced with N/A values. Concentrations for SPARC, FN1, and ALDH1A1 were outside of reliable detection limits for all strains and were excluded from analysis. To generate the principal component analysis (PCA) plot in R v.4.1.2 (33) (**Supplementary Fig. 4B**), analytes MMP3, CXCL1, and IL8 were also excluded from analysis because of N/A values for some samples. GraphPad Prism v.9.3.1 was used to perform paired T-tests and simple linear regressions.

### Bulk RNA-sequencing

Fibroblasts were sorted into CD105$^+$ and CD105$^-$ subpopulations using the markers described above (**Supplementary Fig. 2A**) with an ARIA III cell sorter (BD Biosciences). CD105$^-$ fibroblasts from donor C145 were excluded from downstream analysis as this population represented a relative CD105$^{low}$ group of cells that was not transcriptionally similar to any other CD105$^-$ samples. RNA extraction from the sorted cells was performed using a Quick-RNA Microprep kit with Zymo-Spin IC columns (Zymo Research, catalog no. R1050). Samples were then submitted to COH integrative genomics core for RNA-seq. RNA-seq libraries were prepared using KAPA RNA mRNA HyperPrep Kit (KAPABiosystems, catalog no. KR1352) according to the manufacturer's protocol. Sequencing was performed on an Illumina NovaSeq 6000 with the paired-end read mode of 101 cycles. RNA-seq reads were trimmed to remove sequencing adapters using Trimmomatic (34). The processed reads were mapped back to the human genome (hg19) using TOPHAT2 software (35). HTSeq (36) and RSeQC (37) software packages were applied to generate the count matrices and strand information, respectively, using default parameters. Differential gene expression analysis was performed in R using DESeq2 (38). A vst() transformed DESeq object was used to generate the PCA plot and heatmap (39). Genes labeled on the heatmap are the top 30 genes differentially expressed in CD105$^+$ vs. CD105$^-$ fibroblasts ordered by adjusted p-value with a minimum expression of 100 normalized count values. A lfcShrink() transformed DESeq object was used to generate the Enhancedvolcano plot (40) that was labeled with significantly upregulated genes related to the Gene Ontology (GO) term "negative regulation of immune system" along with *ENG* and *CD248*. topGO (41) was used to perform GO term enrichment analysis on genes significantly upregulated (log2FoldChange > 1 & baseMean > 1 & padj < 0.05) in CD105$^+$ compared to CD105$^-$ fibroblasts. Pathways with Kolmogorov-Smirnov values less than 0.05 were considered significantly enriched (**Supplementary Fig. 2B**). Further pathway analysis was performed with clusterProfiler (42) on differentially expressed genes (log2FoldChange > or < 0 & padj < 0.05) ranked by log2FoldChange. Ingenuity pathway analysis (IPA) (43) of significantly differentially expressed genes (log2FoldChange > 1 or < -1 & baseMean > 1 & padj < 0.05) between *BRCA1*$^{mut/+}$ CD105$^+$ and younger AR CD105$^+$ fibroblasts was analyzed with the use of QIAGEN IPA.

## scRNA-seq analysis

For visualization of gene expression at single-cell resolution and single cell gene set enrichment analysis (scGSEA), three data sets were used: both Kumar et al. 2023 (21) and Reed et al. 2024 (24) were downloaded from CELLXGENE (44) as Seurat objects (45) and GSE161529 (46) was provided as a Seurat object by the group of Dr. Andrea Bild. Downstream analysis was performed on fibroblasts and myeloid cells from the Kumar et al. dataset; stroma and fibroblasts from the Reed et al. dataset, and fibroblasts from the Pal et al. dataset. Data processing and analysis was performed in R. ssCustomize() was used to visualize the expression of single genes and the CD105 signature top 30 differentially expressed genes (> 100 mean normalized count and ordered by adjusted P value) between CD105$^+$ and CD105$^-$ fibroblasts (**Supplementary Table 3**) on UMAPs from each dataset. scGSEA was performed using the escape R package (47) that utilizes UCell (48) to execute and visualize GSEA across individual cells.

Comparisons between cell types and groups were subjected to the Kruskal-Wallis test with post-hoc Wilcoxon to determine p-values. (21). The Kruskal-Wallis test with post-hoc Wilcoxon was used to compare mean signatures between groups and revealed that nearly every comparison was extremely significantly different ($p < 0.0001$), but that was likely a reflection of very small differences amplified by massive sample sizes, thus masking actual biological differences (Fig. 6A**&B, Supplementary Fig. 6B-E, G-I**). For this reason, p-values were not plotted and instead stars represent standardized mean difference calculations. Standardized mean differences were calculated by subtracting the means of two groups and dividing by the pooled standard deviation not accounting for sample size of each group (49). CellChat (v.1.1.3) (50) was applied with default parameters to myeloid cells and fibroblasts from Kumar et al. to infer differential strength and number of interactions between cells differing in menopausal status.

## Immunofluorescent staining in cultured fibroblasts

Fibroblasts were seeded onto glass coverslips in 24-well plates (Greiner Cellstar, 662160) at a density of 5,263 cells/$cm^2$ in M87A + 5%FBS. Fibroblasts were grown until close to confluence, fixed with ice cold methanol:acetone for 15 minutes, blocked with immunofluorescence (IF) blocking buffer (5% donor goat serum (R&D Systems, S13150) and 0.1% Triton X-100 (Sigma-Aldrich T8787) in phosphate-buffered saline (PBS)) for 1 hour at room temperature, incubated with a staining solution with antibodies targeting CD105 and ACTA2 (alpha smooth muscle actin) (**Supplementary Table 4**) overnight at 4°C on a rotating shaker, coverslips were washed with PBS, incubated with a secondary staining solution with Hoechst for 2 hours at room temperature, washed with PBS, and mounted using Fluoromount-G (Electron Microscopy Sciences, 17984). Multiple representative images were acquired for each coverslip using a Nikon Eclipse Ti2. Coverslips only stained with secondary antibodies served as controls and were used to set exposure times for image acquisition. CellProfiler (51) was used to determine the mean intensity of ACTA2 and CD105 for each fibroblast from each strain. Mean intensity values were plotted using geom_pointdensity in R after rescaling values between 0 and 1.

## Immunofluorescent staining of tissue sections

Formalin fixed paraffin-embedded (FFPE) tissue sections were deparaffinized and rehydrated by washing slides in xylene 3 times for 3 minutes each, dunking slides 20 times in 3 different 100% ethanol coplin jars, dunking slides 20 times in 2 different 95% ethanol coplin jars, and removing ethanol from each slide by rinsing with $diH_2O$. Antigen retrieval was performed by incubating the slides with citric acid-based antigen unmasking solution (Vector Laboratories, H-3300) at 98°C for 10 minutes; slides were allowed to cool at room temperature until they reached 35°C. Then, sections were washed with PBS for 5 min. Samples were incubated in IF blocking buffer at room temperature for 1 hour. Primary antibodies targeting CD248, K14 (also listed as KRT14, myoepithelial marker), K19 (also listed as KRT19, luminal marker) (**Supplementary Table 4**) were diluted into IF blocking buffer and incubated with the samples overnight. Samples were washed three times with PBS, stained with appropriate fluorophore-conjugated secondary antibodies (**Supplementary Table 4**) and Hoechst stain 33342 (**Supplementary Table 4**) for 2

hours at room temperature. Slides were washed 3 times with PBS at room temperature and then coverslips were mounted using Fluoromount-G. Multiple representative images of ducts and lobes were acquired using a Nikon Eclipse Ti2 or a Zeiss LSM 700. Fiji (52) was used to select lobular and ductal structures based on keratin staining patterns: lobular structures were characterized by sporadic staining of the luminal marker K19 and sparse staining of myoepithelial marker K14; ductal structures were identified by presence of bilayered epithelium with $K19^+$ luminal cells lining the lumen surrounded by $K14^+$ myoepithelial cells(53). The mean fluorescence intensity of CD248 of a selected region was divided by the average mean fluorescence intensity of the background to determine the relative intensity of CD248 staining for a particular structure. Mann-Whitney test was used to determine whether differences between groups were significant.

## Immune cell source

Day of leukapheresis, PBMCs were isolated by density gradient centrifugation over Ficoll-Paque (GE Healthcare) followed by multiple washes with PBS containing 1 mM EDTA (PBS-EDTA, Cellgro). Monocytes were isolated from freshly collected PBMCs using CD14 antibody-conjugated microbeads and magnetic columns (Miltenyi Biotec, 130-050-201) according to the manufacturer's protocol. $CD14^+$ and CD14– fractions were frozen in CryoStor CS5 (StemCell Technologies) in liquid nitrogen until initiation of coculture experiments.

## Macrophage differentiation

Primary human pro- and anti-inflammatory macrophages were differentiated and polarized as previously described (54, 55). Briefly, frozen human monocytes ($CD14^+$) were thawed and cultured in cytokine-containing RPMI (Lonza) + 10% FBS (heat-inactivated, gamma-irradiated Hyclone) for 8 days in 6-well plates (104,200 cells/$cm^2$ seeding density) (CytoOne). To differentiate pro-inflammatory macrophages, cells were cultured with GM-CSF (BioLegend, 572903). The media was changed once after 4 days to media containing GM-CSF, IFN-γ (BioLegend, 570202), LPS (Sigma-Aldrich, L3012) and IL-6 (BioLegend, 570804). To differentiate anti-inflammatory macrophages, cells were cultured with M-CSF (BioLegend, 574804). The media was changed once after 4 days to media containing M-CSF, IL-4 (BioLegend, 574002), IL-13 (BioLegend, 571102) and IL-6. All cytokines and LPS were used at 20 ng/mL. After another 4 days of differentiation, macrophages were dissociated using 1mM PBS-EDTA, and phenotype was assessed by flow cytometry (CD80, CD206, and CD163; **Supplementary Table 2**) and qPCR (**Supplementary Table 5**) to confirm successful polarization. Cells were counted and used for further studies.

## Cocultures

After 8 days of macrophage differentiation and polarization, sorted (using the protocol outlined in *Flow cytometry and cell sorting*) $CD105^+$ and $CD105^-$ fibroblasts (see **Supplementary Table 1** for strains and passage numbers) were seeded at a ratio of 1:2.5 (fibroblast:macrophage) onto macrophages that had been washed to remove polarization media (Fig. 4A). Cocultures were maintained for 4 days in RPMI +

10%FBS with a media change at day 2. Macrophages cultured without fibroblasts in base media served as controls. At day 4, fibroblasts and macrophages were dissociated using 1mM EDTA and passed through a 100 μm filter to remove cell aggregates that did not dissociate. Macrophages were separated from fibroblasts using a magnetic bead CD45 positive isolation kit (STEMCELL, 100–0105) according to the manufacturer's protocol.

Triculture experiments were conducted in XVIVO 15 (Lonza) + 10%FBS to maximize T cell viability. Following the two-phase differentiation and polarization protocol, macrophages were profiled by flow cytometry (CD80, CD206, and CD163; **Supplementary Table 2**) and counted. 100,000 macrophages were seeded in each well of a 24-well plate (Greiner Cellstar, 662160). 40,000 sorted (using the protocol outlined in *Flow cytometry and cell sorting*) $CD105^+$ and $CD105^-$fibroblasts (C147, passage 4) were counted and seeded onto adherent macrophages. $CD14^-$ cells were thawed and profiled for $CD3^+$ T cell proportion by flow cytometry (**Supplementary Table 2**). The T cell enriched fraction was labeled with CFSE violet (ThermoFisher, C34571) according to the manufacturer's protocol. T cells were then stimulated with CD3/CD28 Dynabead (ThermoFisher, 11161D) at a ratio of 1 bead to 1 T cell. 100,000 labeled, stimulated T cells were then added to the appropriate wells and coculture was maintained for 3 days prior to analysis of T cell proliferation by flow cytometry. Unstimulated T cells served as a negative control and stimulated T cells alone served as the positive control.

## Coculture endpoint analysis

A fraction of macrophages were analyzed by flow cytometry (CD45, CD80, CD163, CD206; **Supplementary Table 2**) and the remaining macrophages were lysed and stored at -80°C until qPCR analysis. Total RNAs were isolated from CD45-enriched macrophages with Quick-RNA Microprep kit (Zymo Research). For qPCR, cDNAs were synthesized with iScirpt reverse transcriptase (Bio-Rad) according to the manufacturer's manual. Quantitative gene-expression analysis was performed by CFX384 real-time PCR (Bio-Rad) with Universal SYBRGreen supermix (Bio-Rad). Data were normalized to *TBP* after calculating relative quantity ($2^{\Delta Cq}$) based on specified control groups. Normalized gene expression was then $\log_2$ transformed. Primers are listed in **Supplementary Table 5**. GraphPad Prism v.9.3.1 was used to perform two-tailed unpaired T-tests.

For macrophage:fibroblast:T cell triculture experiments, T cells in suspension were collected with light pipetting and stained with CD3, CD4, and CD8 targeted antibodies (**Supplementary Table 2**). T cells were gated based on CD3 and either CD4 or CD8 expression. Unstained T cells were used to establish CD3, CD4, and CD8 gates. CFSE-labeled, unstimulated T cells were used to establish the gate for proliferating T cells (**Supplementary Fig. 5G&H**). GraphPad Prism v.9.3.1 was used to perform two-tailed unpaired T-tests.

## Results

## CD105 expression increases in early passage primary fibroblasts from high-risk women

CD105 expression was used to distinguish between interlobular/ductal ($CD105^-$) and lobular ($CD105^+$) fibroblasts present in early passage, primary fibroblasts from 30 women (**Supplementary Fig. 1A**). Peri-epithelial primary fibroblast strains from 20 average risk (AR) women ranging in age from 16 to 70 years were established by isolating and sub-culturing the fibroblasts that migrated from epithelial organoids onto the culture plastic surface, when they were purified by differential trypsinization and then sub-cultured. CD105 expression was measured using flow cytometry. A simple linear regression shows a strong positive correlation between age and proportion of fibroblast population that express CD105 (Fig. 1A). Because $CD105^+$ fibroblasts were found to be enriched in older women, who are more susceptible to breast cancer, and $CD105^+$ fibroblasts have been described as the source of myofibroblast-like cancer associated fibroblasts (myCAFs) that are associated with poor patient outcomes (15), we measured CD105 proportions in another group of women with genetically increased breast cancer susceptibility. Flow cytometry was used to measure CD105 expression in fibroblasts isolated from women with pathogenic *BRCA1* mutations and fibroblasts from these women have significantly (P = 0.026) larger $CD105^+$ populations (mean = 83.9%) when compared to their age-matched AR peers (mean = 64.9%) (Fig. 1B). $CD105^+$ fibroblasts were reported to have a slower growth rate than their $CD105^-$ counterparts (8, 14, 15), and this was found to be true in four strains from different risk groups (Fig. 1C). Taken together, these results indicate that $CD105^+$ fibroblasts are enriched in populations at higher risk for breast cancer.

Early passage fibroblasts were uniformly positive for fibroblast markers CD13 (13, 56), CD109, platelet-derived growth factor receptor alpha (PDGFRα), and platelet-derived growth factor receptor beta (PDGFRβ) (57–60) and mesenchymal stem cell (MSC) markers CD73, CD90, CD166, and protein C receptor (PROCR) (61–63)(**Supplementary Fig. 1C**). A marker of CAFs, podoplanin (PDPN), was the only marker found to vary in expression between the three groups analyzed, as $BRCA1^{mut/+}$ fibroblasts had significantly (P = 0.0016) less PDPN than older AR fibroblasts (**Supplementary Fig. 1C**), which agrees with observations made about CAFs derived from women with *BRCA1* mutations (64).

## $CD105^+$ fibroblasts from women with differences in breast cancer susceptibility differ transcriptionally

To further define differences between $CD105^+$ and $CD105^-$ fibroblasts and fibroblasts from women with differences in breast cancer susceptibility, RNA-sequencing was performed on sorted fibroblasts from 15 individuals (**Supplementary Fig. 2A**). Sequenced samples predictably separate by CD105 expression in a principal component analysis (Fig. 2A). When samples are clustered by the top 30 differentially expressed genes between $CD105^+$ & $CD105^-$ three populations emerge: $CD105^-$ (with specimen 122 $CD105^+$ as the lone outlier in this cluster), $CD105^{middle}$ (C023, 051L, 240, C073, C078, 112, 160, and C051 $CD105^+$), and $CD105^{high}$ (C094, C045, 169, 117, C145, and C147 $CD105^+$) (Fig. 2B). This data further supports the idea that there are grades to CD105 expression and it is notable that the CD105$^{high}$ group is composed primarily of older and *BRCA1*$^{mut/+}$ strains (save for the oldest younger AR strain, 169) (15, 65). A majority of differentially expressed genes between CD105$^{+}$ & CD105$^{-}$ fibroblasts are genes that are upregulated in CD105$^{+}$ fibroblasts. Gene ontology (GO) pathway analysis of the upregulated genes in CD105$^{+}$ fibroblasts relative to CD105$^{-}$ fibroblasts found that the most enriched biological process in CD105$^{+}$ fibroblasts is negative regulation of the immune system (**Supplementary Fig. 2B**). Genes from this pathway that are upregulated in CD105$^{+}$ fibroblasts are labeled on the volcano plot in Fig. 2C along with *ENG* (*CD105*) and *CD248*, which is a proxy marker for CD105$^{+}$ fibroblasts Further pathway level analysis of differentially expressed genes shows that CD105$^{-}$ fibroblasts are enriched for MSigDB hallmark pathways related to proliferation (Fig. 2D **&** Fig. 1C). Genes differentially expressed in CD105$^{+}$ fibroblasts are enriched in pathways related to myogenesis, adipogenesis, angiogenesis, and TGFβ signaling (Fig. 2D). CD105$^{+}$ fibroblasts were also enriched for a host of pathways related to inflammation including: inflammatory response, IL6 signaling, IFNγ response, IFNα response, and TNFα signaling which indicates that these fibroblasts are associated with a chronically inflamed microenvironment (Fig. 2D).

Observing transcriptional differences between CD105$^{+}$ and CD105$^{-}$ fibroblasts is not novel (8, 65, 66), and we next determined whether CD105$^{+}$ fibroblast populations differed between individuals with differences in breast cancer susceptibility (Fig. 2E). The greatest number of differentially expressed genes are between CD105$^{+}$ and CD105$^{-}$ fibroblasts from individuals in the same risk group; however, a more interesting observation was that CD105$^{+}$ fibroblasts from women who carry *BRCA1* mutations are transcriptionally distinct from CD105$^{+}$ fibroblasts from their age-matched AR counterparts (Fig. 2E). Ingenuity pathway analysis (IPA) showed that the top pathways enriched in CD105$^{+}$ *BRCA1*$^{mut/+}$ fibroblasts were related to fibrosis, autoimmune diseases, and cytokine storm signaling (Fig. 2F). This result further strengthens the connection between CD105$^{+}$ fibroblasts and immune modulation and suggests that fibroblasts from women with *BRCA1* mutations may be especially prone to fibrosis and immune cell interactions. CD105$^{+}$ fibroblasts have a unique transcriptional signature that suggests they have characteristics of myofibroblasts, are capable of transdifferentiating, contribute to inflammation, and may interact with immune populations.

## CD105$^{+}$ fibroblasts are capable of adipogenesis and secrete macrophage polarization factors

Because CD105$^{+}$ fibroblasts express markers enriched on adipose stem cells *e.g.* CD13, CD73, CD90, PDGFRα, and PDGFRβ (**Supplementary Fig. 1C**) (67, 68), have upregulated expression of genes related to adipogenesis, like *MEDAG* and *EBF1* (69)(Fig. 2B**&D**), and have previously been shown to exhibit adipogenic potential like mesenchymal stem cells (MSCs)(8, 70) we quantified the adipogenic potential of CD105$^{+}$ fibroblasts from 15 individuals. CD105$^{+}$ and CD105$^{-}$ fibroblasts were grown in DMEM/F12 + 10% FBS until they were subconfluent when media was switched to an adipogenic media for 2 weeks.

Following culture in adipogenic media, cells were fixed and neutral lipids were stained with an oil red O (ORO) solution. ORO stained oil fat vacuoles are prominent in $CD105^+$ fibroblasts treated with adipogenic media compared to the background signal in other conditions (Fig. 3A). $CD105^+$ fibroblasts have significantly greater optical density (OD) 492nm values when compared to $CD105^-$ fibroblasts indicating greater adipogenic potential (Fig. 3B). No difference in adipogenic potential between $CD105^+$ fibroblasts from different risk groups was observed and within each group there was large variability in adipogenic potential between $CD105^+$ fibroblasts (**Supplementary Fig. 3A&B**).

Though not statistically significant, the fact that the top three strains with the greatest adipogenic potential are from women older than 55 years of age suggests that fibroblasts may contribute to increased adiposity of the breast with age (71, 72) (**Supplementary Fig. 3B**). Adipogenesis is one assay to identify MSC populations along with osteogenesis and chondrogenesis (61). $CD105^+$ fibroblasts did not exhibit ability to differentiate into osteoblasts when compared to a MSC positive control as measured by Alizarin-red staining (data not shown).

RNA sequencing also suggested that $CD105^+$ fibroblasts exhibit a myofibroblast like state with enrichment of the “Myogenesis” term (Fig. 2D). Despite enrichment of this term, $CD105^+$ fibroblasts regardless of donor did not reliably coexpress alpha smooth muscle actin (ACTA2), a marker of myofibroblasts (73) (Fig. 3C **& Supplementary Fig. 3C**). There was variability between strains in numbers of $ACTA2^+$ fibroblasts present in culture and a trend towards greater expression of ACTA2 in cells that had lower levels of CD105 expression (**Supplementary Fig. 3C**).

The discordance between mRNA and protein abundance makes it challenging to define essential components of a niche using RNA-sequencing alone (74). To combat this issue, a majority of the analytes selected to be measured using a custom Luminex panel were the protein products of significantly differentially expressed genes between $CD105^+$ and $CD105^-$ fibroblasts (75). The remaining analytes measured were selected because they were either associated with immune modulation or had been previously reported to be associated with $BRCA1^{mut/+}$ fibroblasts (e.g. MMP3) (76). Six of the 19 analytes measured within detectable ranges differed in abundance between $CD105^+$ and $CD105^-$ fibroblasts (Fig. 3D). Strains clustered by CD105 expression when analyzed by PCA as was observed using RNA (**Supplementary Fig. 4A&B;** Fig. 2A). All 6 differentially secreted proteins by $CD105^+$ fibroblasts are involved in either the establishment of an immunosuppressive microenvironment or the direct polarization of macrophages towards an anti-inflammatory phenotype (Fig. 3D) (77–85). Closer consideration of the effects of proteins produced by $CD105^+$ fibroblasts suggest a tension in maintaining the $CD105^+$ state. While POSTN can increase the proliferation and ACTA2 expression of myofibroblasts (86), both DKK1 and CCL2 inhibit myofibroblast differentiation and may explain why the sequencing data does not match ACTA2 expression in cultured cells (Fig. 2D, Fig. 3C) (87, 88). A similar story emerges when contrasting TSP2, a potent inhibitor of angiogenesis (81), with IGFBP-2, a protein that supports angiogenesis and the migration of endothelial cells (83). The 6 proteins with increased abundance in the supernatant derived from CD105$^+$ fibroblasts compared to CD105$^-$ fibroblasts had significant correlation between RNA and protein level data (Fig. 3E). For the proteins that did not differ in abundance between fibroblast subtypes, only 3 of 13 had a significant correlation between RNA and protein expression (**Supplementary Fig. 4C&D**). The Luminex data suggest CD105$^+$ fibroblasts have immunosuppressive characteristics, may influence myofibroblast differentiation, and may be involved in remodeling vasculature.

## Fibroblast subtypes influence immune cell polarization and function

We next assessed whether CD105$^+$ fibroblasts established an immunosuppressive microenvironment by influencing the polarization of macrophages. Primary cocultures were established leveraging a previously published protocol for differentiating pro- and anti-inflammatory macrophages from primary monocytes (Fig. 4A) (55). After differentiation/polarization (Fig. 4A), pro-inflammatory macrophages express CD80 and anti-inflammatory macrophages uniformly express CD206 and CD163 (**Supplementary Fig. 5A**). After 4 days of direct coculture between differentially polarized macrophages and sorted fibroblasts (**Supplementary Fig. 5B**), CD80 expression is completely lost in all conditions (data not shown). CD206 is significantly induced and suppressed in pro- and anti-inflammatory macrophages, respectively, when cocultured with fibroblasts (Fig. 4B, **left**). There is a trend towards increased CD163 expression in pro-inflammatory macrophages with fibroblast coculture and a significant increase in CD163 expression in anti-inflammatory macrophages cocultured with CD105$^+$ fibroblasts compared to anti-inflammatory macrophages after 4 days without polarization media (Fig. 4B, **right**).

After 4 days of coculture with fibroblast subtypes, macrophages were isolated using magnetic bead enrichment for CD45. This method yields a pure macrophage population with good viability (**Supplementary Fig. 5C&D**). Expression of pro-inflammatory genes were then assessed by qPCR (Fig. 4C**)**. *TNFα* expression decreases in pro-inflammatory macrophages after 4 days of culture but is significantly further decreased by coculture with CD105$^+$ fibroblasts. *IL1β* expression also decreases in culture for pro-inflammatory macrophages; however, anti-inflammatory macrophages cocultured with fibroblasts significantly increase the expression of this pro-inflammatory gene. *CXCL1* expression is maintained at baseline (D0) levels by fibroblast coculture in pro-inflammatory macrophages and induced in anti-inflammatory macrophages cocultured with fibroblasts. *IL6* expression also increases relative to monoculture in both macrophage subtypes cocultured with fibroblasts. Immediate and transient expression of these pro-inflammatory cytokines is essential for response to environmental stressors, and chronic expression can contribute to immune exhaustion and disease states (89, 90). Expression of anti-inflammatory genes was also measured (Fig. 4D). *IL10* expression is increased in pro-inflammatory macrophages after 4 days regardless of culture condition, but expression is significantly increased in anti-inflammatory macrophages cocultured with fibroblasts. *CD206* increases in expression in pro-inflammatory macrophages cocultured with fibroblasts to the same level as freshly polarized anti-inflammatory macrophages which agrees with protein level expression (Fig. 4B, **left)**. CD105$^-$ fibroblasts significantly increase the expression of *CD206* in anti-inflammatory macrophages which according to new research, may actually improve adaptive immune surveillance of tissue (91). *CD163* expression increases in expression in pro-inflammatory macrophages cocultured with fibroblasts to a level that is similar to D4 anti-inflammatory macrophages in monoculture. Surprisingly, when considering protein level expression (Fig. 4B, **right)**, *CD163* expression decreases in anti-inflammatory macrophages in coculture with fibroblasts. *FN1* expression is lost in culture in anti-inflammatory macrophages, but fibroblast coculture can induce expression in pro-inflammatory macrophages to levels akin to freshly polarized anti-inflammatory macrophages and expression is maintained in anti-inflammatory macrophages cocultured with fibroblasts. Fibroblast coculture increased expression of genes relevant to tumor-associated macrophages (Fig. 4E**)** (92–94). *CXCL12* expression was significantly increased in pro-inflammatory macrophages cocultured with $CD105^{-}$ fibroblasts and fibroblast culture regardless of CD105 expression increased *CXCL12* expression in anti-inflammatory macrophages. The expression of the cognate receptor for *CXCL12, CXCR4*, only increased in anti-inflammatory macrophages cocultured with fibroblasts. *IL33* expression was induced in anti-inflammatory macrophages cocultured specifically with $CD105^{+}$ fibroblasts. This cytokine has been associated with anti-inflammatory macrophages contributing to Th2 immune responses as well as the expansion and immunosuppressive function of myeloid-derived suppressor cells (MDSCs) in the tumor microenvironment (95, 96). We find that these peri-epithelial fibroblasts maintain and enhance macrophage polarization.

A more effective readout of a macrophage's polarization state is its functional ability to inhibit T cell proliferation. Primary tricultures were established between CD3/CD8 stimulated T cells, polarized macrophages, and sorted fibroblasts (Fig. 5A). Triculture did not influence the composition of $CD4^{+}$ and $CD8^{+}$ T cells in culture (Fig. 5B). Proliferative clusters were observed when T cells were stimulated with CD3/CD28 beads and when stimulated T cells were cultured with pro-inflammatory macrophages (**Supplementary Fig. 5E**). T cells labeled with a CellTrace dye were removed from culture after 3 days and proliferation was measured by quantifying dilution of dye. Most cells in suspension were $CD3^{+}$ (**Supplementary Fig. 5F**). Around 40% of both $CD4^{+}$ and $CD8^{+}$ T cells are proliferating after 3 days in culture (Fig. 5C**&D**). Both pro- and anti-inflammatory macrophages suppress the proliferation of stimulated T cells in culture, but the suppressive effect anti-inflammatory macrophages have on proliferation of T cell subtypes is greater than that of pro-inflammatory macrophages (Fig. 5C**&D**). There is a non-significant trend of decreased proliferation in T cells cultured with pro-inflammatory macrophages and $CD105^{+}$ fibroblasts compared to T cells in combination with $CD105^{-}$ fibroblasts (Fig. 5C**&D**). Anti-inflammatory macrophages dramatically suppress the proliferation of T cells, which is apparent when viewing representative histograms of CellTrace dilution (**Supplementary Fig. 5G&H**). An enhancement of this immunosuppressive phenotype would be difficult to detect, but $CD105^{-}$ fibroblasts reduce the ability of anti-inflammatory macrophages to suppress $CD4^{+}$ T cell proliferation relative to anti-inflammatory macrophages alone (Fig. 5C). $CD105^{+}$ fibroblasts maintain the ability of anti-inflammatory macrophages to suppress $CD4^{+}$ T cell proliferation (Fig. 5C). $CD105^{+}$ fibroblasts maintain the immunosuppressive effects of anti-inflammatory macrophages and coax pro-inflammatory macrophages towards a more anti-inflammatory phenotype.

*The CD105$^{+}$ signature identifies macrophage-interacting fibroblasts that increase in postmenopausal women.*

To assess the *in vivo* relevance of the conclusions drawn from analyzing cultured CD105$^{+}$ fibroblasts, we performed single-cell gene set enrichment analysis (scGSEA) using UCell (48) on publicly available scRNA-seq datasets from human breast tissue (21, 24, 46). The top 30 differentially expressed genes between CD105$^{+}$ and CD105$^{-}$ fibroblasts (Fig. 2B) comprise the CD105$^{+}$ fibroblast signature (Fig. 6A**&B, Supplementary Fig. 6A-I**) that is more effective at identifying specific cell types and subtypes than single genes (*ENG & CD248*) (**Supplementary Fig. 6A&F**). Enrichment of the CD105 signature is greatest in the fibro-prematrix subtype of fibroblasts from Kumar et al., which through spatial transcriptomics were found to be enriched around TDLUs (Fig. 6A). In this same dataset, the CD105 signature was also enriched in fibroblasts from postmenopausal women and women with dense breasts (Fig. 6B, **Supplementary Fig. 6C**). Also in this dataset, the signature was decreased in fibroblasts from underweight women and women of Asian descent (**Supplementary Fig. 6B&D**). A technical observation from the preparation of single cell suspensions for the Kumar et al. dataset is that the CD105 signature is most enriched in fibroblasts from tissues dissociated for the shortest period, and enrichment decreases with increasing dissociation time (**Supplementary Fig. 6E**). Leveraging another dataset that has tissue from both AR and *BRCA1* mutation carriers, the CD105 signature was enriched in fibroblast subtypes FB1 and FB2 which were both found to increase with age and parity (**Supplementary Fig. 6F**) (24). Considering CD105 expression in flow data (Fig. 1A), it was surprising that fibroblasts from *BRCA1* mutation carriers were not found to be enriched for the CD105 signature and in another dataset (46), *BRCA1*$^{mut/+}$ fibroblasts had decreased enrichment of the signature (**Supplementary Fig. 6H&I**). Cell:cell interactions between fibroblast and innate immune cell subtypes were investigated using CellChat (50). The number and strength of interactions between M2 (anti-inflammatory) macrophages and fibro-prematrix fibroblasts increase in postmenopausal women with a concurrent decrease in the interactions between M1 (pro-inflammatory) macrophages and fibro-prematix fibroblasts in these women (Fig. 6C). All prior scRNA-seq analysis was performed on preneoplastic fibroblasts. Leveraging a publicly available dataset comprised of CAFs from different breast cancer subtypes (46), the CD105 signature was found to be most enriched in CAFs associated with triple-negative breast cancer (TNBC) (**Supplementary Fig. 6I**). TNBCs are often characterized by abundant infiltration of tumor associated macrophages that exhibit immunosuppressive effects (97, 98). Further research is needed to determine if CD105$^{+}$ CAFs in TNBC contribute to this aspect of the tumor microenvironment. scRNA-seq analysis revealed the CD105 signature established from cultured fibroblasts can identify a fibroblast subpopulation enriched around TDLUs, that increases in postmenopausal women and has increased communication with anti-inflammatory macrophages.

Continuing our efforts to relate observations made in primary fibroblasts back to the tissue, we sought to measure the expression of CD105 in tissue sections. Unfortunately, because the CD105 epitope is sensitive to formalin fixation, we were unable to measure expression in FFPE sections that come from some of the same donors as the fibroblast strains analyzed. To remedy this, CD248, which is highly upregulated in $CD105^+$ fibroblasts (Fig. 2C), and has previously been described as a proxy marker for CD105 (14) with comparable enrichment in TDLUs as CD105 (8), was used to identify intralobular fibroblasts *in situ*. Lobules and ducts were identified in tissue sections by morphology and keratin expression in epithelial cells (**Supplementary Fig. 6J**) and relative intensity of CD248 was then measured in 12 specimens (**Supplementary Fig. 6K**). Stromal cells surrounding ducts with clear delineation between myoepithelial ($K14^+$) and luminal ($K19^+$) cells had lower expression of CD248 compared to lobules that are characterized by sporadic K19 and K14 expression (Fig. 6D **left panel**). CD248 expression was significantly higher in lobules compared to ducts, but did not differ in expression in either structure between AR and *BRCA1* mutation carriers (Fig. 6D **right panel**).

## Discussion

We previously introduced the concept of accelerated aging in the epithelial compartment of women who possessed clinically relevant *BRCA1* mutations (99, 100). It was not known whether this phenomenon extended beyond the parenchyma. In this manuscript we demonstrate that in younger women, the distribution of $CD105^+$ and $CD105^-$ fibroblasts is about an equal ratio, but with age, this distribution skews towards $CD105^+$ fibroblasts. We observed a similar trend in fibroblasts from younger women with *BRCA1* mutations that is consistent with the idea of accelerated aging of the stroma. $CD105^+$ fibroblasts were found to be a stable population in culture and are enriched for genes related to negative regulation of the immune system, angiogenesis, myogenesis, and adipogenesis. There are transcriptional differences between $CD105^+$ fibroblasts from average risk and $BRCA1^{mut/+}$ donors – $BRCA1^{mut/+}$ $CD105^+$ fibroblasts are enriched for genes related to fibrosis and chronic inflammatory disorders. An essential aspect of the results we report is the experimental validation of the significance of pathways related to adipogenesis and negative regulation of the immune system.

$CD105^+$ fibroblast-specific effects on macrophages include: increasing expression of CD163 in anti-inflammatory macrophages, suppressing *TNFα* expression in pro-inflammatory macrophages, increasing *IL33* expression in anti-inflammatory macrophages, and maintaining anti-inflammatory macrophage-mediated suppression of CD4 T cell proliferation. The association between $CD105^+$ fibroblasts and anti-inflammatory macrophages extended beyond the dish as fibroblast subpopulations from postmenopausal women found to be enriched for the $CD105^+$ signature had increased interactions with anti-inflammatory (M2) macrophages and fewer interactions with pro-inflammatory (M1) macrophages when compared to their premenopausal counterparts. The aged stroma may increase susceptibility to breast cancer as it becomes more adipogenic and immunosuppressive (Fig. 6E).

Reed et al. reported changes to the proportions of populations that were conserved between women with WT and mutant *BRCA1* genotypes. A unique feature of the *BRCA1*$^{mut/+}$ microenvironment was the increased infiltration of CD8$^+$ T cells and the increased expression of *PDL1* on luminal cells and macrophages that would combine to describe an exhausted immune microenvironment ripe for immune escape (24). Increased median cell density of CD8$^+$ and CD4$^+$ T cells in the normal/benign breast lobules of *BRCA* (*BRCA1 or BRCA2*) mutation carriers compared to average risk women was reported (101). In that study population comprised primarily of women < 55 years of age, lobules of *BRCA* mutation carriers not only had increased numbers of T cells compared to average risk counterparts, but also CD68$^+$ macrophages and CD11c$^+$ dendritic cells. When aging is taken into consideration, mean density of CD3$^+$ T cells decline with age only in high-risk women (22). Increased immune infiltration in women carrying *BRCA1* mutations was attributed to increased mutational burden leading to the formation of neoantigens (102). In the present study, cultured CD105$^+$ fibroblasts were shown to influence macrophage polarization and maintain macrophage's ability to suppress T cell proliferation. Future work should directly address how CD105$^+$ fibroblasts may directly recruit T cells through CCL2 (Fig. 2C) or in combination with macrophages due to the expression of *CXCL12* (Fig. 4E).

*BRCA1*-associated cancers, especially TNBC, are characterized by increased infiltration of tumor-promoting immunosuppressive macrophages, CD4$^+$ $T_{regs}$, and exhausted T cells (20, 103, 104). How CAFs contribute to this immune microenvironment is an active field of study. CD105$^+$ CAFs in the murine pancreas were found to be tumor permissive whereas CD105$^-$ CAFs were able to support anti-tumor immunity *in vivo* mediated through T and B cells (66). Beyond immunosuppression, CD105$^+$ CAFs have been shown to differentiate cancer cells and increase their metastatic potential in models of cancer in the prostate and colon (65, 105). In the breast, CD105$^+$ CAFs are associated with metastatic recurrence and increased tumor size (9) as well as being prognostic markers for bone metastasis in early breast cancer patients (10). Even though the CD105$^+$ fibroblast signature is enriched in TNBC CAFs and not in CAFs from other subtypes (**Supplementary Fig. 6I**), CD105$^+$ CAFs are still relevant to the other breast cancer subtypes that are more associated with aging. Morsing et al. originally described the division of inter- from intralobular fibroblasts using both CD105 and CD26 to mark subpopulations (8). As reported in this manuscript, they also described CD105$^+$/CD26$^-$ fibroblasts to be slow growing, transcriptionally similar to tumor stroma (myofibroblast-like), have adipogenic and osteogenic differentiation capacity, and support luminal outgrowth which is dependent on TGFβ activity (8, 14). Building on this foundation, they found that CD105$^-$/CD26$^+$ fibroblasts support myoepithelial differentiation and bipotent progenitor cell activity (14). Most recently, CD105 expression was found to mark myCAFs and absence of expression in iCAFs from ER$^+$ breast cancers (15). Collectively, these findings highlight the complexity of fibroblast heterogeneity in shaping the tumor microenvironment and emphasize the need to target specific fibroblast populations, especially in aging-related breast cancers.

Defining fibroblast subtypes is an important pursuit because it allows for greater understanding about normal niches, precancerous niches, and the cell of origin for CAFs. A population of PROCR$^+$/

ZEB1$^+$/PDGFRα$^+$ (PZP) fibroblasts are enriched in African American breast tissues (106). Like the CD105$^+$/CD26$^-$ fibroblasts, these PZP fibroblasts were capable of adipogenic and osteogenic differentiation and when cocultured with epithelial cells allowed for luminal progenitor basalization (106). This fibroblast subpopulation was found to secrete factors also related to polarization of anti-inflammatory macrophages like CXCL12, HGF, CSF1, and POSTN (107, 108). Where these researchers found heterogeneity in PROCR and PDGFRα expression, the fibroblasts analyzed in this manuscript had ubiquitous expression of both markers. This is the great challenge of defining subsets of fibroblasts in culture and efforts should be made to define populations first in tissue using spatial transcriptomics and multi-omics approaches. A more holistic understanding of fibroblasts in native states must begin in tissue and can be supplemented by investigation in culture.

A strength of this manuscript is the number of strains used in each experiment. Only through profiling so many strains from donors differing in age and *BRCA1* status can the variability between individuals be appreciated and trends emerge. Transcriptional profiling of cells can provide valuable insights into pathways that are differentially activated in one cell type vs. another; however, sequencing results can often produce more candidate genes than are experimentally tractable. Top gene candidates found to be differentially expressed between CD105$^+$ and CD105$^-$ fibroblasts were explored at the protein level either through ACTA2 staining or through Luminex assay. Six of the 23 analytes measured were found to be significantly differentially secreted by CD105$^+$ fibroblasts when compared to matched CD105$^-$ fibroblasts. These six analytes all were associated with the establishment of an immunosuppressive niche and this manuscript explored the interaction between fibroblast subtypes in the breast and immune cells which up to this point had yet to be attempted in culture. Cocultures between CAFs and immune cells are well-trodden, but the novelty of this manuscript is that it explores the interactions between normal fibroblasts and immune cells from healthy donors. Rather than relying on non-physiological concentrations of pleiotropic cytokines, direct cocultures likely provide a more realistic insight into how stromal cells may influence immune subpopulations. The confusion in interpreting the results of the fibroblast:macrophage coculture experiments highlights the importance of moving beyond a false M1/M2 dichotomy (109). The CD105 phenotype established and explored here in culture identifies a subpopulation of fibroblasts *in vivo* that increase in older women and are enriched around TDLUs.

Should there be further support in the future for the expansion of CD105$^+$ fibroblasts as contributing factors to breast cancer susceptibility, this population or the proteins secreted by these fibroblasts could be directly targeted. Carotuximab, a humanized monoclonal antibody developed to inhibit angiogenesis by targeting CD105, has been used in clinical trials to target CD105$^+$ CAFs in metastatic, castration resistant prostate cancer (110). Far-fetched prevention studies can be imagined that would use this therapeutic to reshape the composition of fibroblasts in the disease-free breast of women at high risk of developing breast cancer. POSTN production was enriched in CD105$^+$ fibroblasts and may be able to be targeted by administration of Warfarin, which was been shown to reduce the production of Gla domain containing proteins (111). Another, preventative therapeutic option is the targeted, short-term administration of ibuprofen or similar NSAIDS to reduce the ability of fibroblasts to establish an immunosuppressive niche (112). Preventing deleterious fibroblast subpopulations from expanding could be more effective than targeting CAFs which likely arise from the same tissue resident fibroblast populations (11, 15).

While sample number is a strength of this paper, it is also paradoxically a weakness. Differences between $CD105^+$ and $CD105^-$ fibroblasts were often subtle. It was only with increased sample number that differences between younger and older luminal cells could be appreciated (113, 114). As the experiments reported here were unable to detect significant differences between younger (n = 6) and older (n = 5) fibroblasts, increasing the sample number may be the only way to reveal differences between younger and aged stromal niches. While fibroblasts had an impact on macrophage polarization, very few differences were observed between how different fibroblast subtypes polarize macrophages. A major challenge during coculture experiments was optimizing media conditions for all cell types. Media conditions that supported macrophage and T cell growth were suboptimal, even inhibitory, for fibroblast proliferation and morphology and vice versa. These challenges with the media may have blunted the true impact of different fibroblast subtypes. Finally, CD105 expression was difficult to detect both in scRNA-seq data as well as in FFPE tissue sections. Low expression of CD105 in fibroblasts in scRNA-seq was remedied by implementing a CD105 signature. CD248 served as a proxy marker for CD105 in tissue; however, $CD248^+$ fibroblasts may not directly overlap with $CD105^+$ fibroblasts which would explain the discrepancy between increased $CD105^+$ fibroblast proportion in cultured fibroblasts from women with *BRCA1* mutations that was not mirrored by CD248 expression in tissue sections.

Future experiments are required to identify the most important factors secreted by fibroblast subtypes related to the establishment of an immunosuppressive niche. These factors may be proteins as analyzed in this paper using an ELISA based assay or these factors may be exosomes or metabolic byproducts. Robust enough phenotypes were not observed to merit downstream mechanistic studies. Similarly challenging experiments should be conducted to understand the impact of fibroblasts on endothelial cells and angiogenesis. Finally, future approaches to define fibroblast heterogeneity should take into consideration menstrual cycle phase (2, 115, 116) and move beyond the use of one or two cell surface markers to define complex and dynamic fibroblast populations.

## Conclusions

$CD105^+$ fibroblasts increase in proportion with age and in women with *BRCA1* mutations. These fibroblasts are enriched for genes related to myCAFs and negative regulation of the immune system and there are transcriptional differences between $BRCA1^{mut/+}$ and $BRCA1^{WT}$ $CD105^+$ fibroblasts. $CD105^+$ fibroblasts secrete proteins related to the establishment of an anti-inflammatory and angiogenic niche. Macrophages cocultured with fibroblasts better maintain their polarization states with $CD105^+$ fibroblasts promoting the expression of CD163 and *IL33* in anti-inflammatory macrophages. The CD105 transcriptional pattern defined in culture is relevant to *in vivo* conditions and identifies a subset of fibroblasts enriched in postmenopausal women that have increased interactions with anti-inflammatory macrophages when compared to fibroblasts derived from premenopausal women. Understanding the function of $CD105^+$ fibroblasts in the preneoplastic niche is essential as $CD105^+$ CAFs are associated with such poor patient outcomes. Coculture models like the ones presented in this manuscript may allow for cell:cell interactions involved in establishing TDLUs that are at high risk of cancer initiation to be studied and manipulated in the dish.

## Abbreviations

Terminal ductal lobular units (TDLUs)

Extracellular matrix (ECM)

Regulatory T cells (Tregs)

Human mammary epithelial cell (HMEC)

Population doublings (PD)

Oil Red O (ORO)

Isopropanol (IPA)

Gene ontology (GO)

Ingenuity pathway analysis (IPA)

Single cell gene set enrichment analysis (scGSEA)

Phosphate-buffered saline (PBS)

Average risk (AR)

Myofibroblast-like cancer associated fibroblasts (myCAFs)

Mesenchymal stem cell (MSC)

Principal component anlysis (PCA)

Optical density (OD)

Myeloid-derived supressor cells (MDSCs)

Triple-negative breast cancer (TNBC)

PROCR+/ZEB1+/PDGFRα+ (PZP)

## Declarations

*Ethics approval and consent to participate*

The present study was approved by the Human Subjects Committee and the Institutional Review Board (IRB) (17185) at City of Hope. Women were consented in person and sequentially; all women signed a City of Hope IRB-approved consent before trial entry. Breast organoids from reduction mammoplasties were prepared at the Lawrence Berkeley National Laboratory (Berkeley, CA) with approved IRB for sample distribution and collection from specific locations. Leukapheresis products (whole blood discard kits) were obtained from consented healthy donors under protocols approved by the City of Hope Internal Review Board.

*Consent for publication*

Not applicable.

*Availability of data and materials*

The authors assert that data supporting the findings of this study not present within the paper or its supplementary information files will be made available upon request. Fibroblast strains are made available either by contacting the corresponding author at City of Hope, or by contacting Dr. Martha Stampfer at http://hmec.lbl.gov.

*Competing interests*

The authors declare that they have no competing interests.

*Funding*

This work was supported by awards from the NIH: EB024989,U01CA244109, R33AG059206, R01EB024989, R01CA237602; Department of Defense/Army Breast Cancer Era of Hope Scholar Award (BC141351); Circle 1500; and City of Hope Center for Cancer and Aging to M.A.L.; and E.G.C. is a CIRM Scholar supported by the California Institute for Regenerative Medicine (EDU4-12772). Research reported in this publication included work performed in the Analytical Cytometry, Analytical Pharmacology, and Integrative Genomics Cores supported by the National Cancer Institute of the National Institutes of Health under grant number P30CA033572. The content is solely the responsibility of the authors and does not necessarily represent the official views of the National Institutes of Health.

*Authors' contributions*

E.G.C. and M.A.L. conceived the study; E.G.C., Y.Y., S.P., and M.A.L. designed the experiments; E.G.C., J.C.L., Y.Y., and J.G. performed the experiments; E.G.C. and Y.Y. analyzed and interpreted the data; E.G.C. wrote the manuscript. All authors read and approved the final manuscript.

Acknowledgements

The authors would like to thank patient advocates Susan Samson and Sany Preto for their consistent input.

## Figures

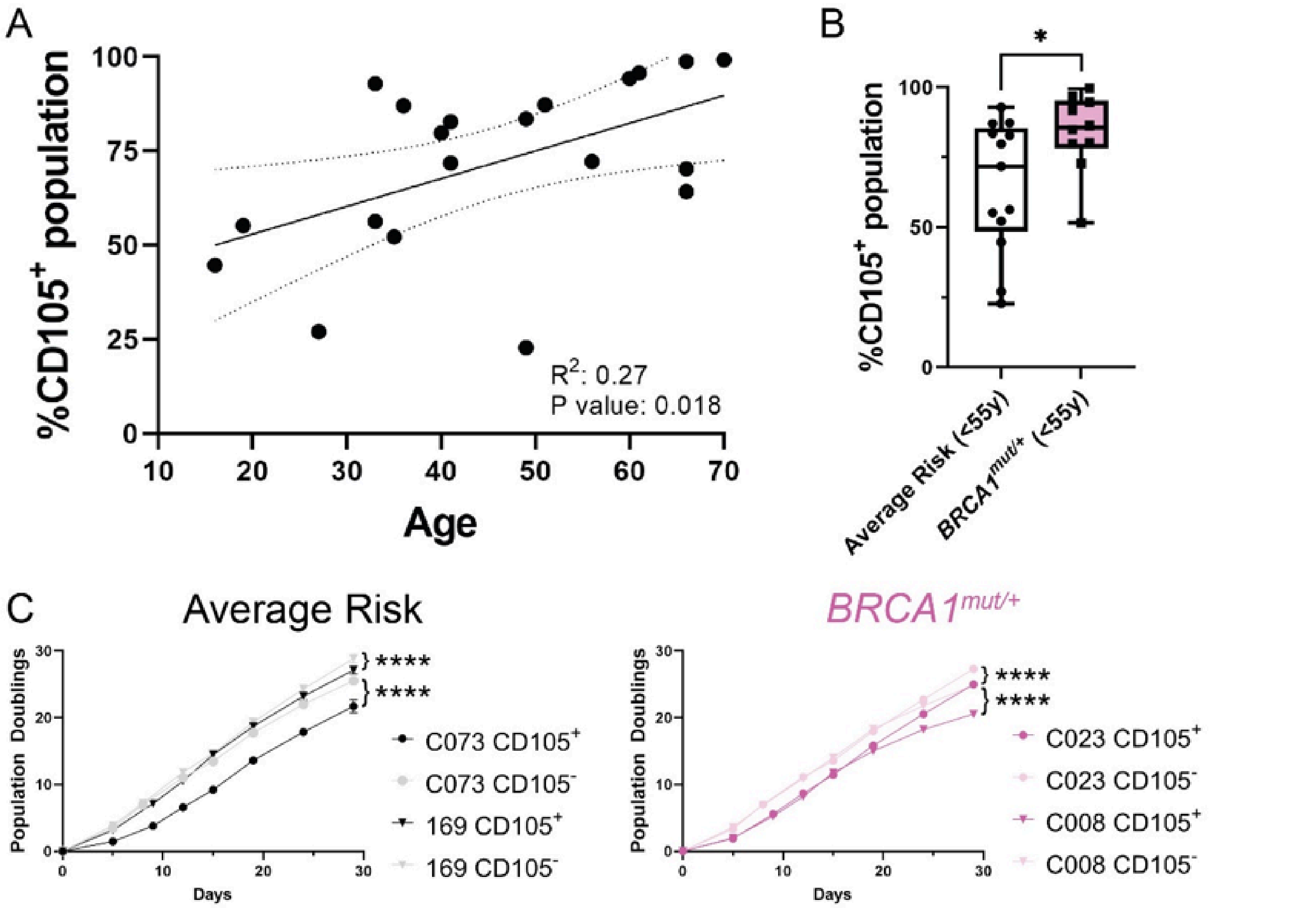

### Figure 1

CD105$^+$ fibroblasts are more abundant in cultures from older women and women with *BRCA1* mutations. **A** Flow cytometry measurement of CD105 proportion in fibroblasts from women ranging from 16 to 70 years of age (n=20); simple linear regression plotted with 95% confidence intervals. **B** Flow cytometry measurement of CD105 proportion in fibroblasts from age-matched women with varying *BRCA1* mutational status (AR n=13, *BRCA1$^{mut/+}$* n=10); unpaired t test with Welch's correction (P value = 0.0263). **C** Population doublings of CD105$^-$ & CD105$^+$ fibroblasts from women with different *BRCA1* mutational status in serial passage subculture, error bars represent standard deviation, two-way ANOVA (**** = $P < 0.0001$).

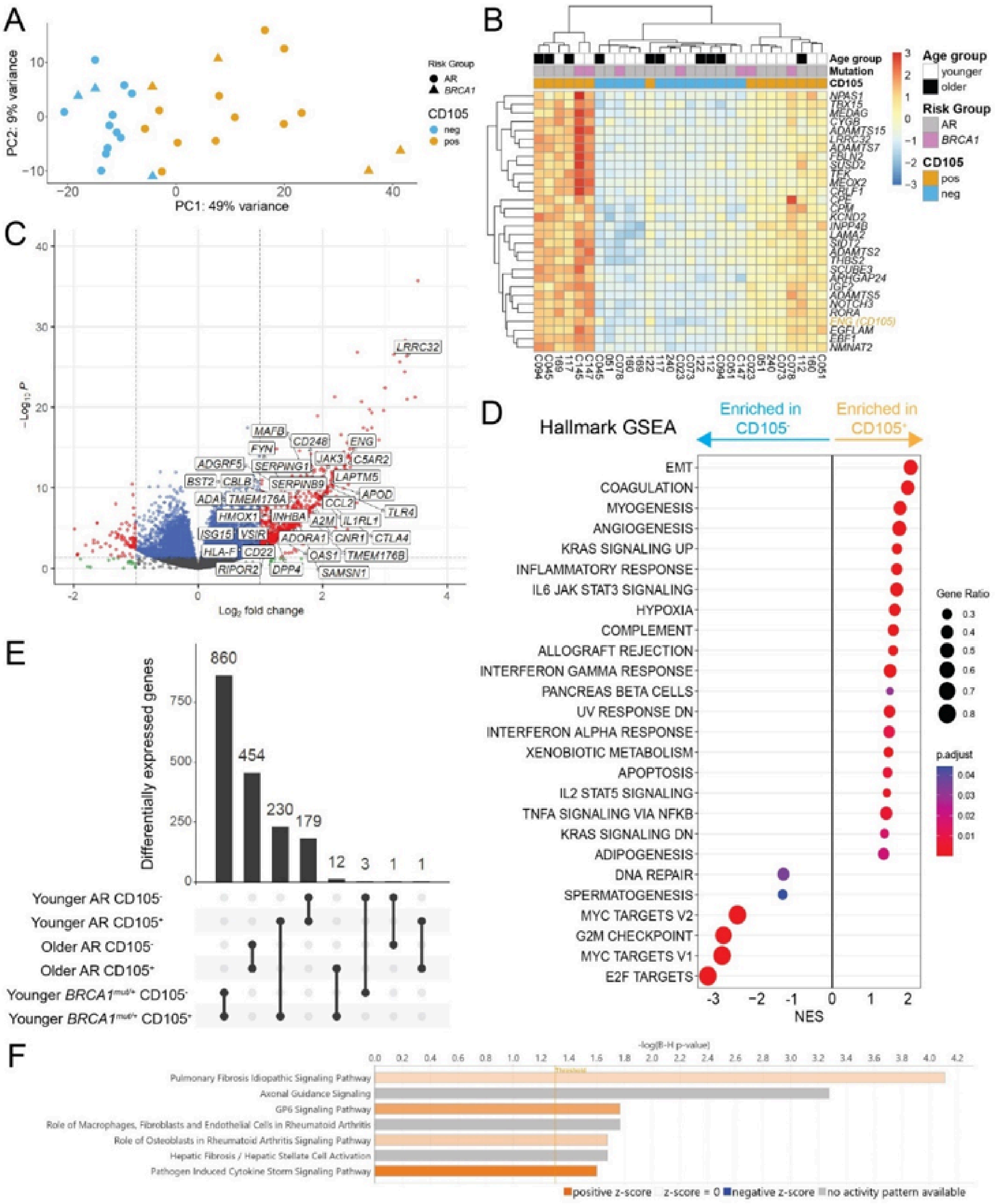

### Figure 2

CD105$^{+}$ fibroblasts are enriched for adipogenesis, angiogenesis, and negative regulation of the immune system pathways. **A** Principal component analysis (PCA) of all expressed genes for the 29 samples analyzed by RNA-seq **B** Heatmap of samples (columns) clustered based on expression of top 30 differentially expressed genes (rows) between CD105$^{+}$ & CD105$^{-}$ fibroblasts; values centered and scaled by row. **C** Volcano plot of differentially expressed genes in CD105$^+$ compared with CD105$^-$ fibroblasts with significantly upregulated genes related to the GO term “negative regulation of immune system” labeled along with *ENG (CD105*) and *CD248*. **D** MSigDB hallmark pathways enriched in CD105$^-$ & CD105$^+$ fibroblasts. **E** Number of differentially expressed genes between fibroblasts from each group. **F** Ingenuity pathway analysis of differentially expressed genes between *BRCA1$^{mut/+}$* CD105$^+$ & younger AR CD105$^+$ fibroblasts. Data were analyzed with the use of QIAGEN IPA (QIAGEN Inc., https://digitalinsights.qiagen.com/IPA).

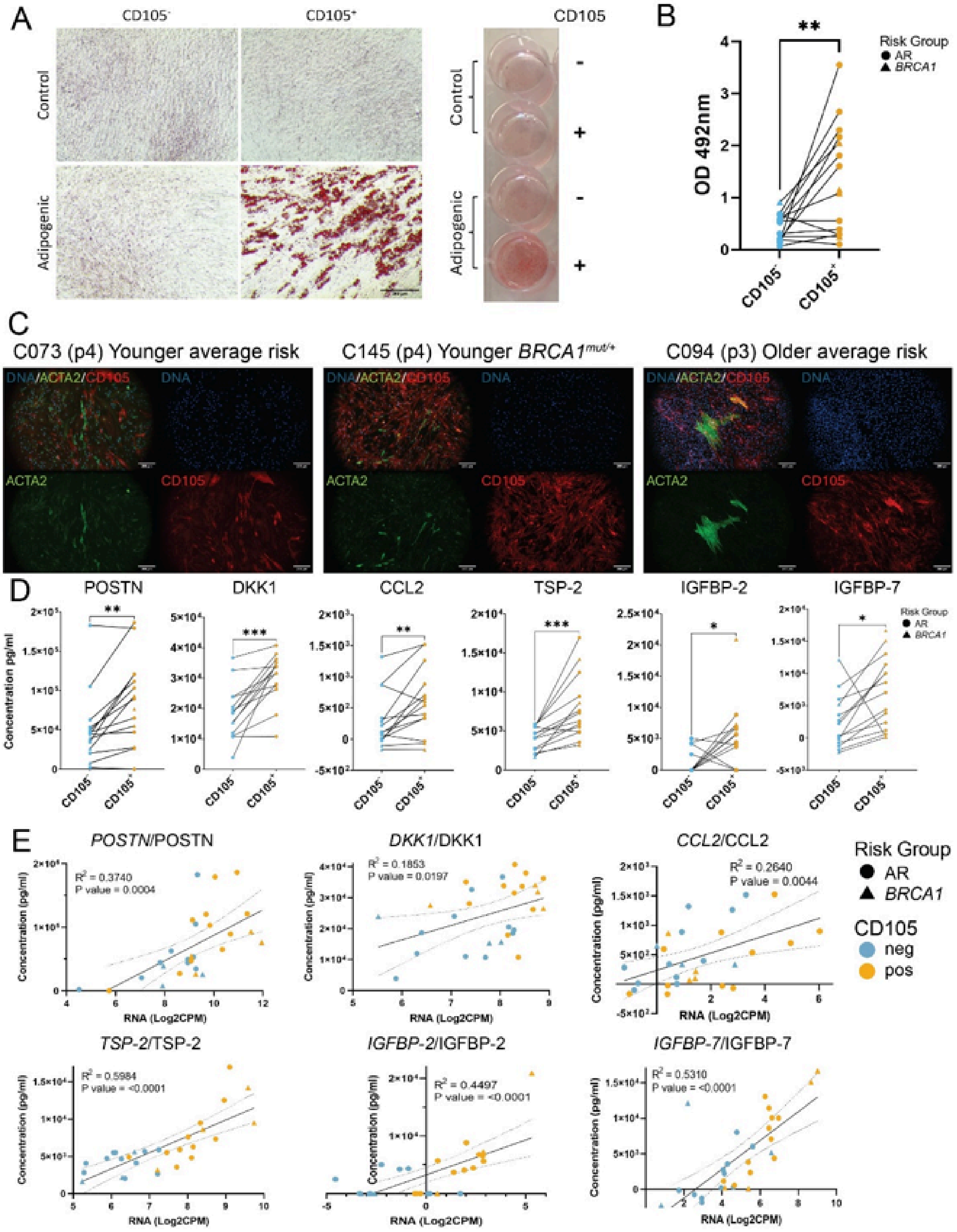

### Figure 3

CD105$^+$ fibroblasts exhibit robust adipogenesis and secrete proteins related to polarization of macrophages and angiogenesis. **A** (left panel) Representative brightfield images (10X objective) from CD105 sorted fibroblasts from strain 169 grown in control or adipogenic media for 2 weeks, fixed, and neutral lipids stained with oil red O (ORO) (scale bar = 200 μm). (right panel) Photograph of wells of a 12-well plate imaged in left panel. **B** Quantification of ORO staining in sorted fibroblasts grown in adipogenic media by measuring OD (optical density) 492nm using a plate-reader spectrophotometer, paired t test (P value = 0.0063). **C** Representative fluorescent images of DNA (Hoechst blue), ACTA2 (green), and CD105 (red) expression in fibroblasts from 3 different donors (scale bar = 200 μm). **D** Concentration of each analyte measured in supernatant of sorted CD105$^+$ and CD105$^-$fibroblasts cultured for 4 days, Paired t test. **E** Correlation between protein and transcript level expression of each analyte in each strain, (CD105$^-$ fibroblasts in blue and CD105$^+$ fibroblasts in orange) simple linear regression plotted with 95% confidence intervals.

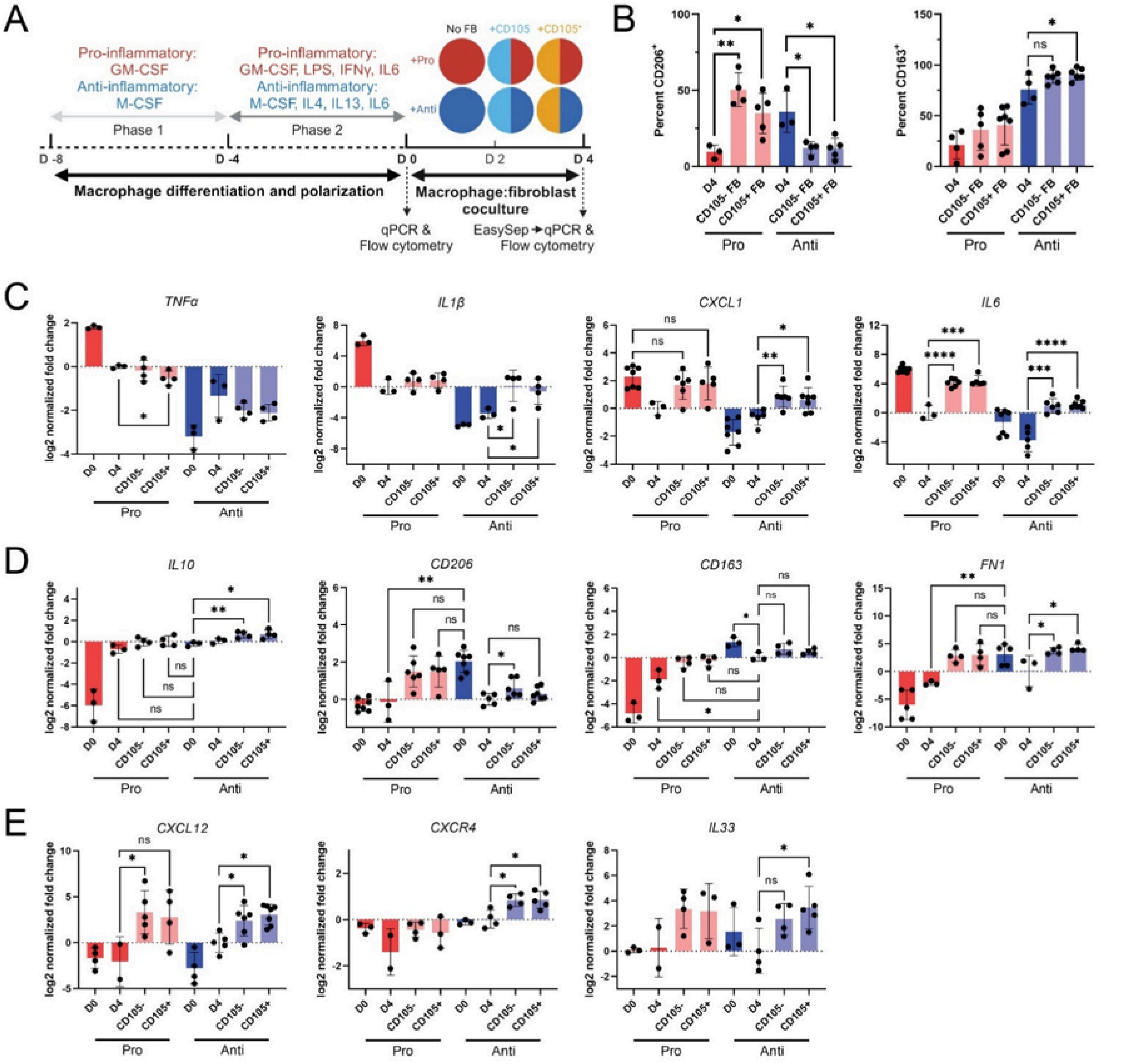

### Figure 4

Fibroblasts influence macrophage polarization. **A** Scheme for macrophage polarization and subsequent fibroblast coculture. **B** Flow cytometry analysis of macrophages enriched for CD45 expression following 4 days of culture without polarization media. **C-E** qPCR analysis of macrophages enriched for CD45 expression following 4 days of culture without polarization media. **C** Pro-inflammatory genes normalized to D4 pro-inflammatory macrophages in monoculture. **D** Anti-inflammatory genes normalized to D4 anti-inflammatory macrophages in monoculture. **E** Tumor-associated macrophage genes normalized to D4 anti-inflammatory macrophages in monoculture, unpaired t tests used to determine significance (ns $P > 0.05$, * $P \leq 0.05$, ** $P \leq 0.01$, *** $P \leq 0.001$, **** $P \leq 0.0001$) and error bars represent standard deviation.

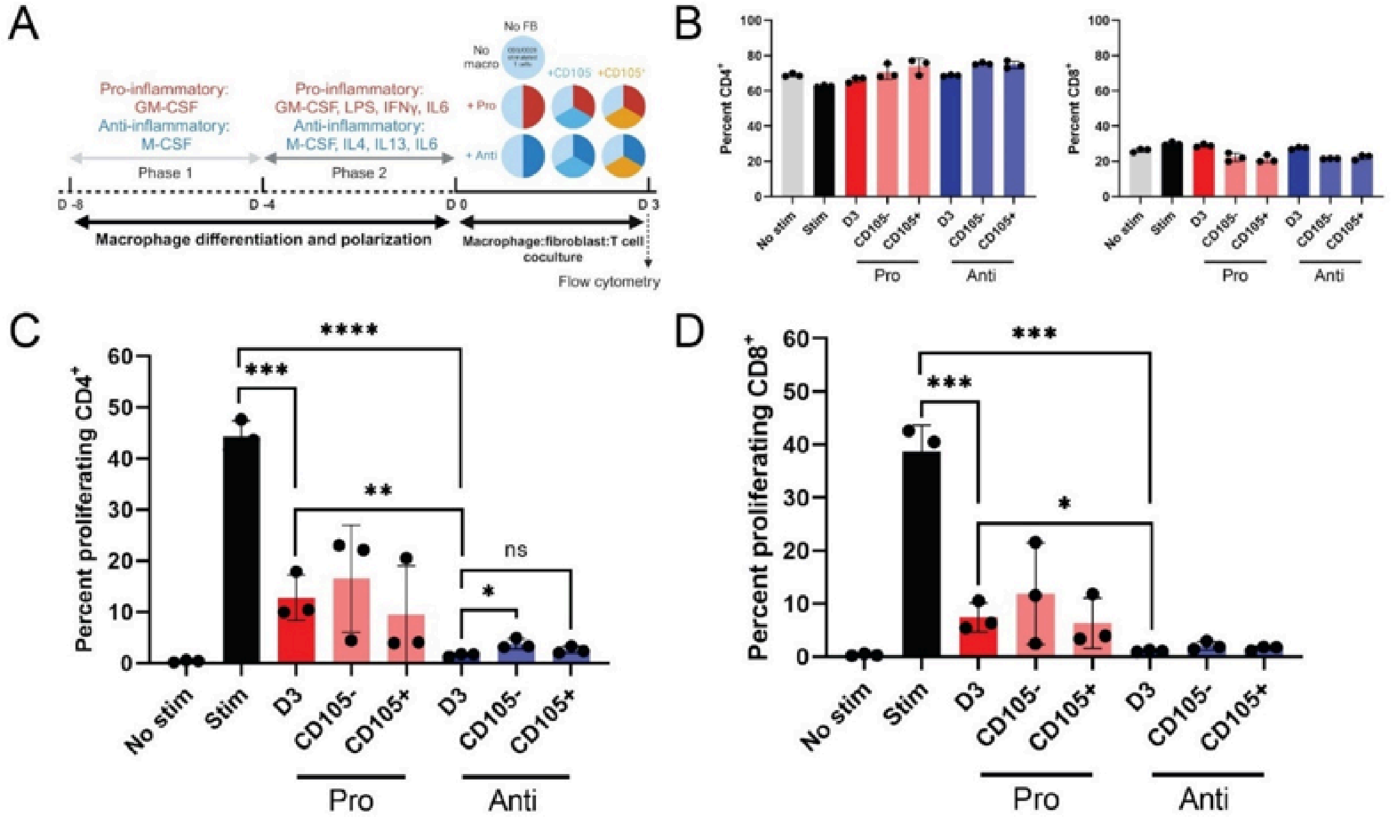

### Figure 5

Fibroblasts have a modest impact on macrophages' ability to inhibit T cell proliferation. **A** Scheme for macrophage polarization and subsequent fibroblast and T cell coculture. **B** T cell subtype composition after 3 days in various coculture conditions: (left panel) percentage $CD3^+CD4^+$ T cells (right panel) percentage $CD3^+CD8^+$ T cells. **C&D** CellTrace was used to label T cells and measure proliferation of T cell subsets after 3 days in various coculture conditions. **C** Percentage proliferating $CD3^+CD4^+$ T cells. **D** Percentage proliferating $CD3^+CD8^+$ T cells, GraphPad Prism v.9.3.1 was used to perform two-tailed unpaired T-tests and error bars represent standard deviation.

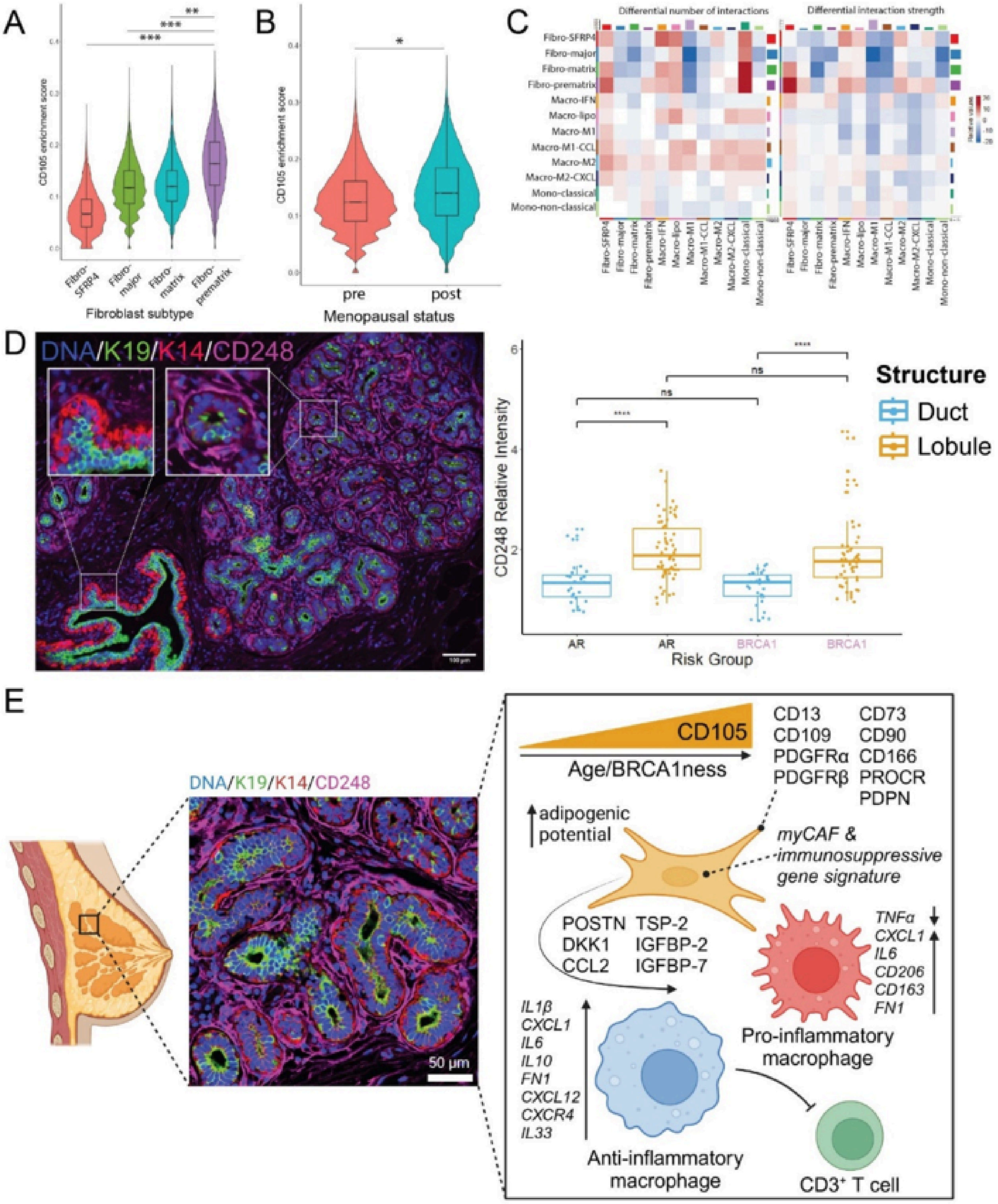

### Figure 6

Contextualizing the CD105$^{+}$ signature. **A&B** scGSEA was performed on fibroblasts from a scRNA-seq dataset (Kumar et al. 2023) using a custom gene list comprised of the top 30 gene differentially expressed in CD105$^{+}$ fibroblasts (**Figure 2B)**; standardized mean difference: small effect size (0.2 – 0.5) *, medium effect size (0.5 – 0.8) **, large effect size (>0.8) ***. **A** Split by fibroblast subtype. **B** Fibroblasts split by menopausal status. **C** CellChat was used to infer number and strength of interactions between different cell subtypes from pre and postmenopausal women (Kumar et al. 2023). **D** (left panel) Representative fluorescent image (20X objective) of DNA (Hoechst blue), KRT19 (K19 green), KRT14 (K14 red), and CD248 (magenta) in a tissue section from donor C073 (scale bar = 100 μm). (right panel) Quantification of CD248 expression in different structures in tissue sections from donors with different *BRCA1* mutational backgrounds, (AR duct n = 28, AR lobule n = 66, HR duct = 30, HR lobule = 52) Mann-Whitney U test (**** = < 0.0001, ns = > 0.05). **E** Graphical summary of $CD105^+$ fibroblasts.

## Supplementary Files

This is a list of supplementary files associated with this preprint. Click to download.

- supplementaryfigures.pdf
- supplementaryinformationtablesfinalrevisions.xlsx
